# Improved Mechanical and Electrochemical Properties of XNBR Dielectric Elastomer Actuator by Poly(dopamine) Functionalized Graphene Nano-Sheets

**DOI:** 10.3390/polym11020218

**Published:** 2019-01-27

**Authors:** Dan Yang, Xinxin Kong, Yufeng Ni, Mengnan Ruan, Shuo Huang, Puzhen Shao, Wenli Guo, Liqun Zhang

**Affiliations:** 1 Department of Material Science and Engineering, Beijing Institute of Petrochemical Technology, Beijing 102617, China; 5120131575@bipt.edu.cn (X.K.); 2018520047@bipt.edu.cn (Y.N.); 1120170209@mail.nankai.edu.cn (S.H.); shaopuzhen@bipt.edu.cn (P.S.); guowl@bipt.edu.cn (W.G.); 2 Beijing Key Lab of Special Elastomeric Composite Materials, Beijing 102617, China; ruanmengnan@bipt.edu.cn; 3 Department of Materials Science and Engineering, Beijing University of Chemical Technology, Beijing 100029, China

**Keywords:** dielectric elastomer actuator, dopamine, graphene nano-sheets, electrochemical properties, latex mixing

## Abstract

In this work, graphene nano-sheets (GNS) functionalized with poly(dopamine) (PDA) (denoted as GNS-PDA) were dispersed in a carboxylated nitrile butadiene rubber (XNBR) matrix to obtain excellent dielectric composites via latex mixing. Because hydrogen bonds were formed between –COOH groups of XNBR and phenolic hydroxyl groups of PDA, the encapsulation of GNS-PDA around XNBR latex particles was achieved, and led to a segregated network structure of filler formed in the GNS-PDA/XNBR composite. Thus, the XNBR composite filled with GNS-PDA showed improved filler dispersion, enhanced dielectric constant and dielectric strength, and decreased conductivity compared with the XNBR composite filled with pristine GNS. Finally, the GNS-PDA/XNBR composite displayed an actuated strain of 2.4% at 18 kV/mm, and this actuated strain was much larger than that of pure XNBR (1.3%) at the same electric field. This simple, environmentally friendly, low-cost, and effective method provides a promising route for obtaining a high-performance dielectric elastomer with improved mechanical and electrochemical properties.

## 1. Introduction

**Figure 1:**
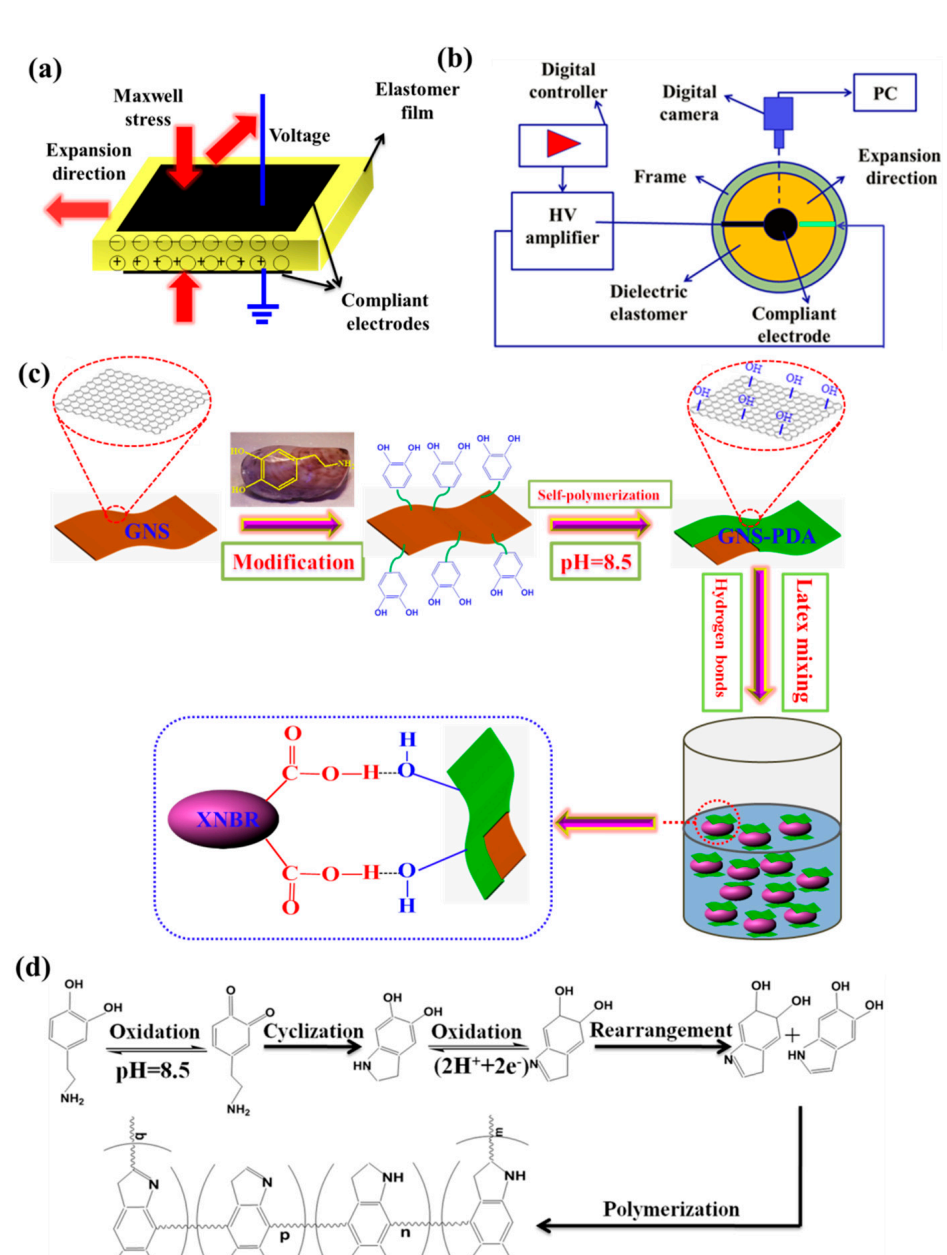
Schematic diagram of (**a**) dielectric elastomer actuator, (**b**) electromechanical testing method, (**c**) preparation of GNS-PDA nano-sheets and the GNS-PDA/XNBR composite; (**d**) Possible mechanism of dopamine oxidative self-polymerization.

Electro-active polymers (EAPs) have attracted ever-increasing attention in recent years because they can mimic human muscles [1]. Dielectric elastomers (DEs) have emerged as frontrunners among all EAP materials because of their compelling properties such as rapid response, high energy densities, large strains, and light weight [2,3]. A dielectric elastomer actuator (DEA) consists of an insulating elastomer film sandwiched between two stretchable electrodes and can expand and contract by applying an external voltage (Figure 1a). As a result, DEAs are of great interest for broad applications, such as soft robots, artifical muscles, adaptive optical devices, and energy harvesters [4]. However, DEAs require a high driving voltage (several kilovolts), which largely limits their practical application because of safety risks and the cost of devices.

The mechanism of DEAs is mainly demonstrated by electrostatic forces, driven by the coulombic force between positive and negative charges. When an external voltage is loaded into the two compliant electrodes, the thickness strain (*S_z_*) of a DEA is determined by the ratio of Maxwell stress (*P* = ε_0_ε_r_(*V*/*Z*)^2^) to the elastic modulus (*Y*) of the DE film, where ε_0_ and ε_r_ are the dielectric constant of vacuum and DE film, respectively, *V* is the driving voltage, and *Z* is the thickness of the DE film. From the mechanism of DEAs, we can find a method for decreasing the driving voltage of a DEA is to reduce the thickness of the DE films and to coat them with stretchable electrodes with a low *Y*_e_·*t*_e_ value, where *Y*_e_ and *t*_e_ are the Young’s modulus and the thickness of the electrode, respectively [5]. Shea et al. [6] used a pad-printing method to produce micrometer-thick silicone DE membranes and achieved a lateral actuation strain of 7.5% at only 245 V. In addition, the driving voltage can also be reduced by improving the electromechanical sensitivity (*β =* ε_r_/*Y*) with an increase in ε_r_ and/or decrease in *Y* [7]. In terms of stress, a low elastic modulus which results from adding a polar solvent or plasticizer into the elastomer matrix is not advantageous, because of the accompanying poor mechanical properties and long-term performance disability [8]. 

Improving the dielectric contant of dielectric materials is a desirable way to obtain large force and strain outputs, and to reduce the driving voltage. Many researchers have focused on increasing the dielectric constant of DEs. The main method to do this is to process ‘two-phase’ composites, including high-dielectric-constant ceramic particles/polymer and conductive filler/polymer composites [9]. Compared with inorganic ceramic particle/polymer composites, a small amount of conductive filler dispersed in a polymeric matrix leads to a sharp increase in the dielectric constant because of the percolation phenomenon [10]. However, there are two challenging issues for preparing conductive filler/polymer composites with a high dielectric constant. One is that the decreased dielectric breakdown strength and increased dielectric loss are usually accompanied by increase in dielectric constant in the composites, leading to limited applications and energy dissipation [11]. Another is that composite preparation conditions should be carefully controlled to ensure that the conductive filler is uniformly dispersed in the polymeric matrix. However, it is difficult to carry out because of the large different surface energy between the conductive filler and polymeric matrix, which causes the conductive filler to gather spontaneously together to form aggregations, that enhance the local probability of dielectric failure or propagation of discharge cascade [12]. 

Graphene nano-sheets (GNS) are novel two-dimensional nano-materials and have attracted enormous interest as a dielectric filler to improve electromechanical properties of DEAs [13]. However, many GNS aggregations easily form in composites because of weak interfacial adhesion between the inorganic phase and organic phase or gravity sedimentation of GNS during preparation [14]. Modifying graphene by grafting it with organic groups, such as SO_3_H [15], alkyl amines [16], and organic isocyanate groups [17], is an effective method for improving the dispersibility and interactivity between graphene and the polymeric matrix. However, these methods require sophisticated polymerization techniques that severely restrict the wide practical application of DEAs. Recently, poly(dopamine) (PDA) films can be synthesized from an aqueous solution of dopamine at a weakly alkaline pH via self-polymerization and have attracted much attention. A large number of amino groups and quinone groups on PDA films causes PDA to show astonishing adhesion to all types of materials and provides versatile potential for secondary functionalization [18]. In fact, many studies have been devoted to preparing graphene-PDA hybrids. Zheng et al. [19] prepared a PDA-reduced graphene oxide (GO) nanocomposites for the simultaneous determination of two isomers of phenolic compounds of hydroquinone and catechol. Zhao et al. [20] synthesized two-dimensional PDA/GO composites and used them as an adsorbent for removing radioactive uranium (VI). Ning et al. [21] coated GO with PDA (denoted as PDA-GO) and used it to adjust the electromechanical properties of carboxylated nitrile butadiene rubber (XNBR) at a low percolation threshold. 

In this work, we improved mechanical and electromechanical properties of XNBR by adding PDA-functionalized GNS (denoted as GNS-PDA) using a bio-inspired method, because the PDA benzene ring can form π-π stacking interactions with GNS. XNBR had a relatively high dielectric constant (>10), which is attributed to strong polar groups, and was used as the dielectric elastomer material. GNS-PDA nano-sheets were introduced into the XNBR matrix through latex mixing. Encapsulation of GNS-PDA nano-sheets around XNBR latex particles is expected to form via hydrogen bonds between –COOH groups of XNBR and phenolic hydroxyl groups of GNS-PDA during latex mixing. The morphology, mechanical properties, and electromechanical properties of XNBR composites filled with GNS before and after modification with PDA were thoroughly investigated.

## 2. Experimental

### 2.1. Raw Materials

XNBR rubber latex with a total solid of about 45% (Nipol LX550L) and a diameter of about 1–2 μm was bought from Nippon Zeon Corp. (Tokyo, Japan). GNS with an average thickness of 4.4 nm (Figure S1) and an average lateral size of 0.5 μm was provided by Beijing Carbon Century Technology Co., Ltd., (Beijing, China). Dopamine (DA) and tris(hydroxymethyl)-amino-methane (Tris) were supplied by Alfa Aesar Co., (Ward Hill, Massachusetts, USA). The crosslinking agent dicumyl peroxide (DCP) was provided by Beijing Chemical Plant (Beijing, China).

### 2.2. Modification of GNS by PDA Self-Oxidative Polymerization

GNS (100 mg) was dispersed into 100 mL of distilled water via ultrasonication for 1 h to get a uniform colloidal suspension of GNS. Then, DA (200 mg) and 10 mM Tris (to adjust the pH to 8.5) were mixed into the above solution and under 10 min stirring at 0 °C. After that, the mixed solution was vigorously stirred at 60 °C for 8 h. Last, GNS-PDA was removed by filtration and washed with deionized water three times. Then, the obtained GNS-PDA nano-sheets were dried under reduced pressure in a vacuum oven at 60 °C until the weight remained constant. 

### 2.3. Preparation of XNBR Dielectric Composites Via Latex Mixing

In this work, the content of GNS was 0.5 phr (parts per hundred parts of rubber) (about 0.23 vol %). The preparation process of the dielectric composites is as follows: First, 0.5 phr GNS or GNS-PDA was dispersed in water via ultrasonic for 0.5 h. Then, the GNS or GNS-PDA aqueous suspension and the XNBR latex were mixed together under vigorous stirring for 1 h. Then, the mixture was co-coagulated using 1% calcium chloride solution. The obtained GNS/XNBR or GNS-PDA/XNBR latex suspension was then dried in a vacuum oven overnight at 60 °C to avoid spontaneous gathering of the GNS filler to form aggregates. Second, 2 phr DCP was added to the latex suspension with mechanical mixing on a two-roll mill. Lastly, the prepared mixture was placed in a mold and vulcanized at 25 MPa and 160 °C for the T_90_ determination using a GT-M2000-FA rotorless curemeter (Goteah Testing Machines Inc., Taichung, Taiwan). 

### 2.4. Characterization Methods

X-ray photoelectron spectroscopy (XPS) was used to analyze the surface of the samples. The measurements were carried out on an ESCALAB 250 XPS system (Thermo Electron Corporation, Madison, Wisconsin, USA). Thermogravimetric analysis (TGA) was tested on a TA SDT650 (TA Instruments, New Castle, DE, USA) under a N_2_ atmosphere. An FEI NanoSEM 430 field emission scanning electron microscope (Thermo Fisher Scientific, Hillsboro, OR, USA) was used to analyze the morphologies of the XNBR composites filled with GNS and GNS-PDA. Stress-strain curves of dumbbell-shaped samples were measured using an Instron 3366 tensile apparatus (Instron Corporation, Norwood, MA, USA) at 50 mm min^−1^. The stress for all the samples is engineering stress. The slope of the stress-strain curve at 10% strain was obtained to determine the elastic modulus values of the samples. Dielectric properties were measured using an Alpha-A broadband dielectric spectrometer (Novocontrol, Montabaur, Germany) at room temperature. DC conductivity was measured using an EST 121 resistivity meter (Beijing Huajinghui, Beijing, China). The electromechanical properties of the DEAs were investigated using circular membrane actuators, as described in our previous study [22]. The schematic of electromechanical testing method is shown in Figure 1b. The samples for actuated strain tests had a thickness of 0.15 mm and a diameter of 5 cm. As it is difficult to accurately measure the change in thickness, we instead measured the change in planar area S_p_ to evaluate the electromechanical properties. The biaxial pre-strain in our measurement is 10%. The planar area strain can be calculated according to *S*_p_ = (*A − A*_0_)/*A*_0_ × 100%, where *A* is the actuated planar area and *A*_0_ is the original area. Before measuring the electromechanical properties of DEAs, compliant graphite electrodes were sprayed on the two sides of the DE films using an airbrush. The compliant electrodes were prepared as follows: First, 10 g of Dow Sylgard 184 silicone rubber, 10 g of Dow Corning Corporation 200^®^ fluid, viscosity 60,000 cSt (25 °C), and 3.133 g of EC-300J carbon black were dispersed in 350 mL of n-heptane under ultrasonication for 2 h at 25 °C. Then, 1 g of Dow Sylgard 184 curing agent was added to the above mixture and further ultrasonicated for 0.5 h at 25 °C. After the DE samples were sprayed with the electrodes, they were put into a drying oven at 60 °C for 8 h. The voltages loaded on the electrode area were supplied by an intelligent DC high voltage generator (LAS6030P, Boer High Voltage Power Supply Co. LTD, Suqian, China). Every experimental data point of dielectric properties, mechanical properties, DC conductivity, and actuated strain in this work is the average of the results obtained from at least five samples under the same condition. 

## 3. Results and Discussion

### 3.1. Preparation of GNS-PDA Nano-Sheets

The detailed preparation procedures of GNS-PDA nano-sheets via PDA polymerization are described in Figure 1c. Under alkaline conditions and oxidation, the DA monomer formed a thin coating on the surface of GNS via self-polymerization in buffer solution. A possible dopamine self-polymerization mechanism is shown in Figure 1d. It is believed that the protons in the dihydroxyl group of DA is oxidized at weak alkaline pH, and forms a key intermediate, 5,6-dihydroxyindole (DHI), after a series of oxidation, cyclization, and rearrangement reactions. DHI is further oxidized to the 5,6-indolequinone, which undergoes further polymerization to form PDA [23,24].

**Figure 2:**
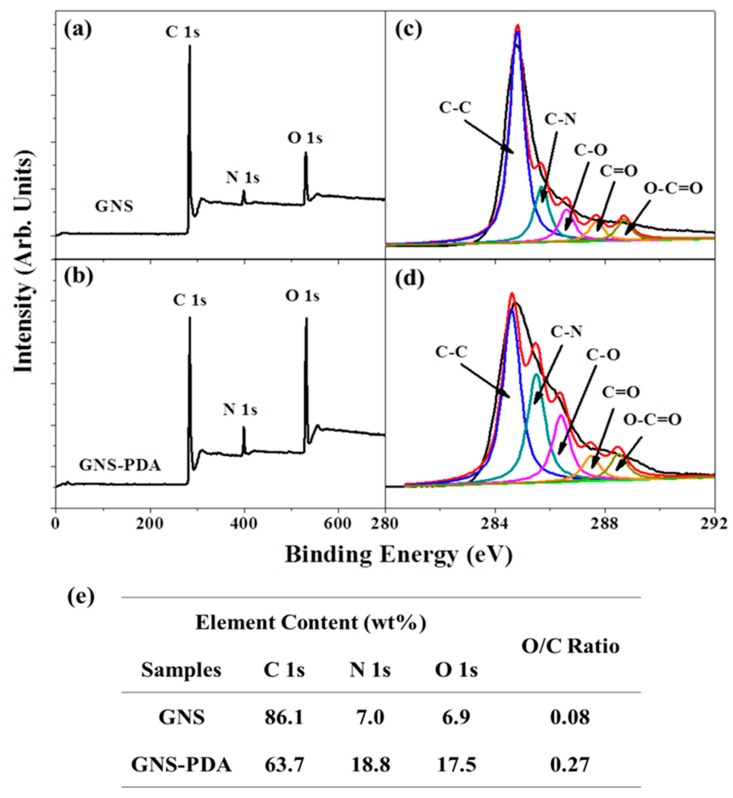
Wide scan XPS of (**a**) GNS and (**b**) GNS-PDA, C 1s core-level spectra of (**c**) GNS and (**d**) GNS-PDA. (**e**) Surface element compositions of GNS and GNS-PDA as determined from by XPS results.

XPS spectra were recorded to analyze the surface of the samples. Wide scan XPS results of GNS and GNS-PDA are shown in Figure 2. As seen in Figure 2a, the wide scan spectrum of GNS displays a peak component of N 1s, and this peak was introduced by the synthesis process. Figure 2b,d shows the C1s core-level spectra of GNS and GNS-PDA, respectively. The deconvoluted C1s XPS spectra of the two samples clearly show five kinds of carbon bonds, including C–C (284.6 eV), C–N (285.5 eV), C–O (286.4 eV), C=O (287.5 eV), and O–C=O (288.5 eV). It is clear that the relative intensity of the C–N peak from GNS-PDA is higher than the C–N peak from GNS, and this demonstrates that PDA adhered successfully onto the GNS surface [21]. In addition, as determined from XPS, the C, N, and O concentrations on the surface of GNS were 86.1%, 7.0%, and 6.9%, respectively. However, after deposition of PDA on GNS, the C, N, and O concentrations changed to 63.7%, 18.8%, and 17.5%, respectively (Figure 2e). The O/C ratio of GNS-PDA increases to 0.27, which is much higher than that of GNS (0.08), because of a much greater amount of oxygen in PDA. 

**Figure 3:**
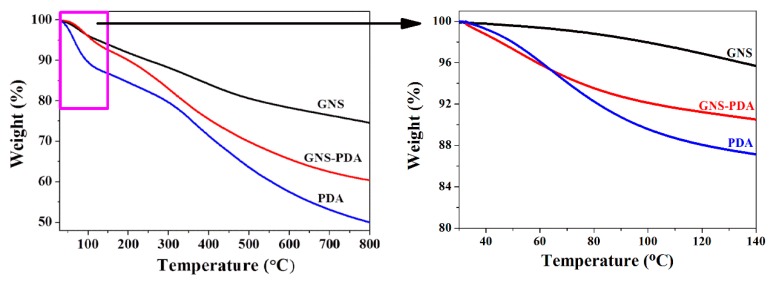
TG curves of GNS, GNS-PDA, and pure PDA.

Figure 3 shows thermal decomposition curves of GNS, GNS-PDA, and pure PDA. In Figure 3, the TGA curve of PDA shows an obvious two-step weight loss process. When the temperature is lower than 140 °C, the weight loss of PDA is attributed to the elimination of water and residual solvent [25]. The weight losses (at 140 °C) of GNS, GNS-PDA, and pure PDA are 4.3%, 9.5%, and 12.85%, respectively, and these values indicate that GNS-PDA has excellent thermal stability under the recommended temperatures with the use of XNBR (<140 °C). When the temperature increases from 150 to 800 °C, the weight loss of PDA is attributed to the partial decomposition of the PDA main chain [26]. It is obvious that the weight loss of GNS-PDA is higher than that of pure PDA and is lower than that of pristine GNS, and this also demonstrates that a thin layer of PDA was successfully coated on the surface of GNS nano-sheets.

### 3.2. Microstructure and Mechanical Properties of XNBR Dielectric Composites

**Figure 4:**
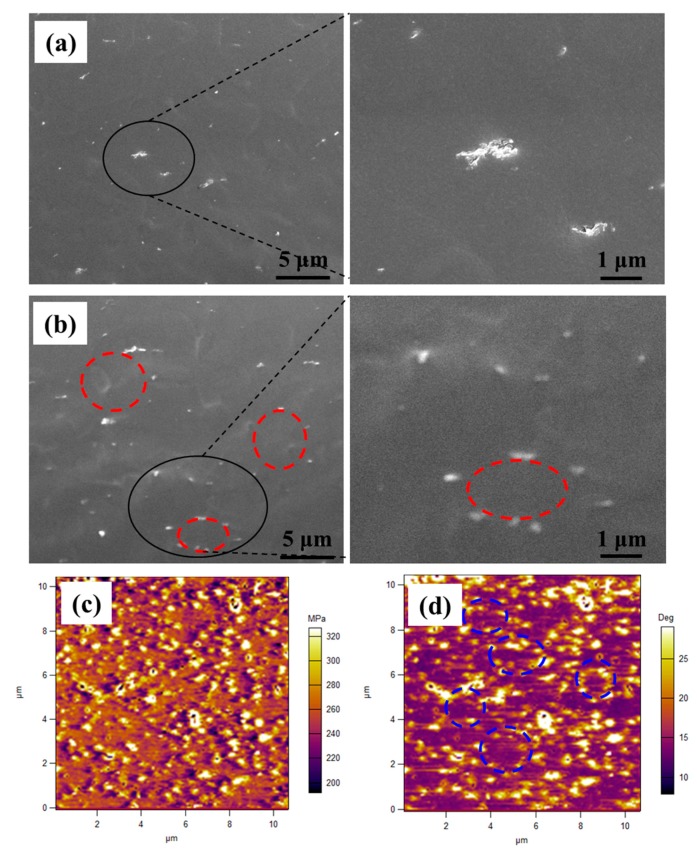
SEM images of (**a**) GNS/XNBR composite and (**b**) GNS-PDA/XNBR composite; AFM images with AM-FM viscoelastic mapping mode of GNS-PDA/XNBR composite: (**c**) moduli image and (**d**) phase image.

Freeze-fractured cross-sectional SEM images of the GNS/XNBR and GNS-PDA/XNBR composites are shown in Figure 4a,b. There are some agglomerations in the GNS/XNBR composite (Figure 4a), which is attributed to the large differences in surface characteristics between the GNS nano-sheets and the XNBR matrix. However, the GNS-PDA nano-sheets are dispersed uniformly around the XNBR latex particles and form a segregated network structure in the GNS-PDA/XNBR composite (Figure 4b), as reported in a previous study [21]. This can be explained by the formation of hydrogen bonds between –COOH groups of XNBR and phenolic hydroxyl groups of GNS-PDA [21]. In addition, the segregated network structure is also observed using an MFP-3D Origin atomic force microscope (AFM) (Oxford Instruments Co., USA) with the AM-FM viscoelastic mapping mode, and the results are shown in Figure 4c,d. The GNS-PDA shows the higher modulus domains periodically, whereas the XNBR matrix shows lower modulus domains aggregated morphology (Figure 4c). Through analyzing the phase image of Figure 4d, it is clear that the GNS-PDA nano-sheets form segregated network structure in the matrix. 

**Figure 5:**
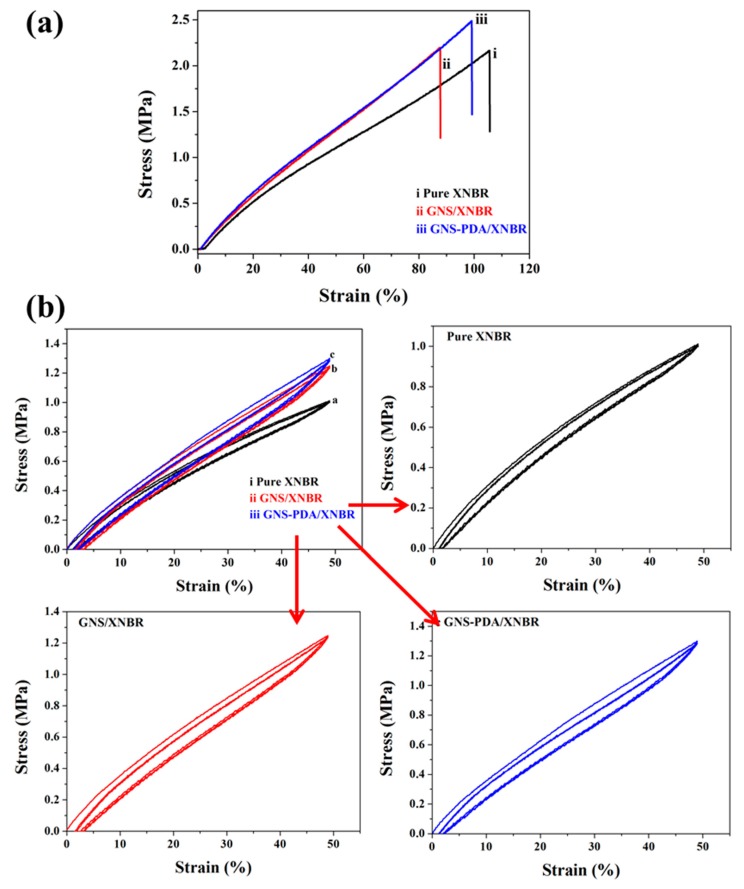
(**a**) Stress-strain curves and (**b**) cyclic stress-strain curves at 50% strain of pure XNBR, GNS/XNBR composite, and GNS-PDA/XNBR composite.

The stress-strain curves of pure XNBR and XNBR composites are displayed in Figure 5a. The GNS-PDA/XNBR composite exhibits a higher tensile strength than that of the pure XNBR and GNS /XNBR composite. The largest tensile strength of GNS-PDA/XNBR composite reaches to 2.5 MPa. The improved tensile strength of GNS-PDA/XNBR composite was attributed to the uniformly dispersion of GNS-PDA nano-sheets in the XNBR matrix with fewer defects and strong interfacial interactions (hydrogen bonds) between the filler and matrix. The strong interfacial interactions lead to restrict slippage of chains and uniform distribution of the external force [27,28], which lead to the GNS-PDA/XNBR composite displays larger elongation at break than that of the GNS/XNBR composite and pure XNBR. Moreover, the cyclic stress-strain curves of pure XNBR, GNS/XNBR composite, and GNS-PDA/XNBR composite are shown in Figure 5b. As seen in Figure 5b, the loading curves of pure XNBR and the XNBR composites show initially stiff responses followed by rollover to more compliant behaviors before stiffening again. The loading curves show residual deformations, which are related to the Mullins effect [29,30]. The residual deformation is thought to be a strain rate-dependent phenomenon that disappears if the deformation rate is small enough [30]. This phenomenon has also been reported in many studies [29,31]. The first loading curve shows a large hysteresis loop, and the following cycles show smaller ones. This effect is referred to as softening behavior [29].

The elastic modulus values of pure XNBR and XNBR composites are shown in Table 1. After the introduction of inorganic GNS, the elastic moduli of XNBR composites are increased. The largest elastic modulus is 3.63 MPa for GNS-PDA/XNBR composite, which is much larger than that of pure XNBR (3.02 MPa) and the GNS/XNBR composite (3.34 MPa) (Table 1). The increased elastic modulus is responsible for the strong interfacial interaction and segregated network structure of the filler. 

### 3.3. Dielectric and Electromechanical Properties of XNBR Dielectric Composites

**Figure 6:**
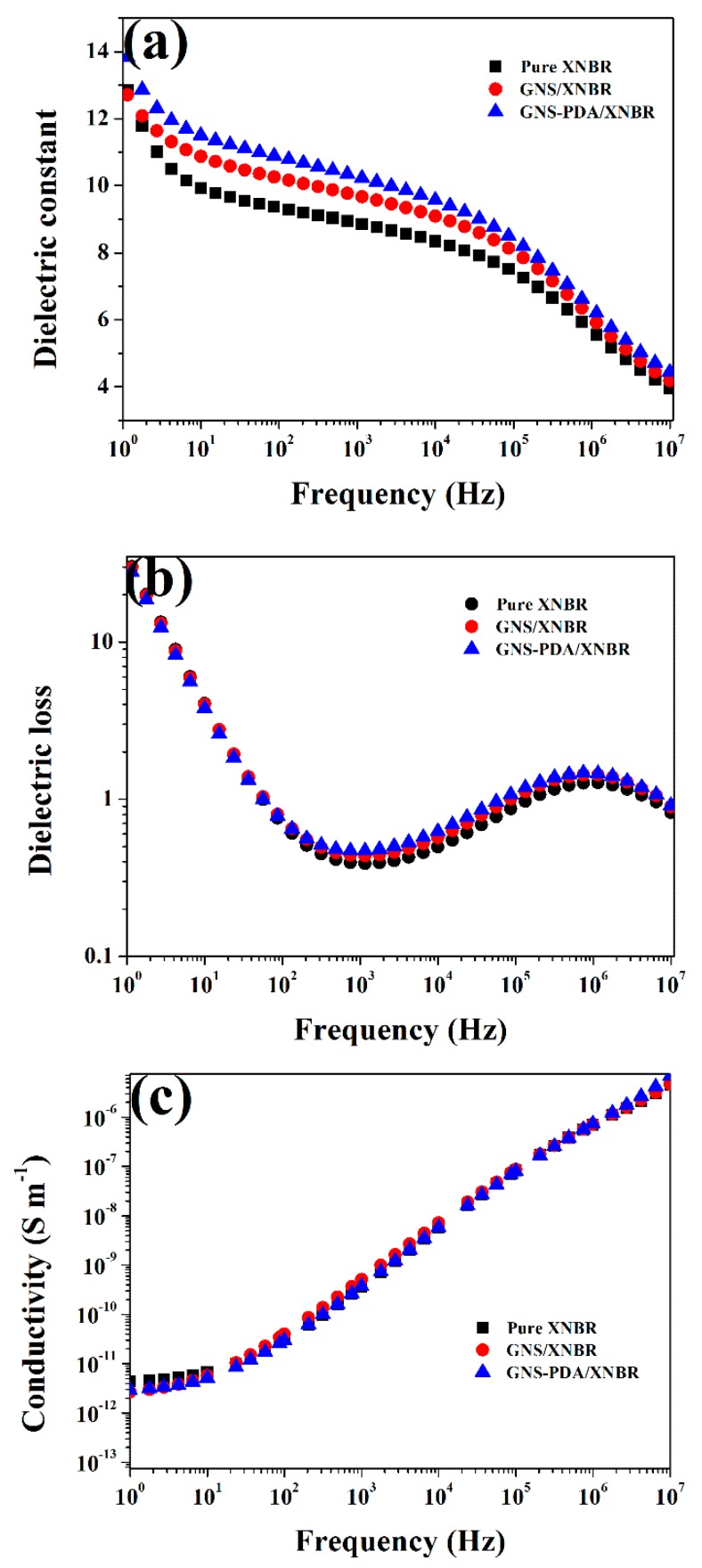
Dependence of (**a**) dielectric constant, (**b**) dielectric loss, and (**c**) AC electrical conductivity on frequency at room temperature for pure XNBR, GNS/XNBR composite, and GNS-PDA/XNBR composite.

The dependence of dielectric properties and AC electrical conductivity of pure XNBR and XNBR composites on frequency is displayed in Figure 6. With an increase in frequency, the dielectric constant decreases for all the samples (Figure 6a), and this means that the dielectric properties are strongly dependent on frequency. This phenomenon is responsible for the dipole polarization of polar groups in XNBR and the interfacial polarization between filler and matrix, which cannot catch up with the changes in electrical field frequency [32]. As seen in Figure 6b, in the low frequency region (10^0^ to 10^2^ Hz), the dielectric loss decreases sharply with an increase in frequency. The explanation for this phenomenon is that the interface polarization (Maxwell-Wagner polarization) at the interface between the XNBR matrix and GNS nano-sheets could not maintain the pace with the increase in frequency [33]. In the high frequency region, the dielectric loss increases again with the increase in frequency, this is ascribed to the dominant contribution of dipolar relaxation. In Figure 6c, the AC conductivity at low frequency is because of the resistive conduction through the bulk composite. However, the AC conductivity appears to be proportional to frequency at high frequency. The explanation for this is that the electrons are sufficiently excited so that they can hop from one conducting cluster to another, leading to an increase in the AC conductivity of XNBR composites [34]. 

In practical application, the frequency used for DEAs is relatively low. In this study, we select 100 Hz to evaluate the dielectric porperties of samples. The dielectric constant at 100 Hz of all the samples is shown in Table 1. Compared with pure XNBR, the dielectric constant of the GNS/XNBR composite and GNS-PDA/XNBR composite is larger because the conductive nature of GNS is helpful for electrical charge moving. In addition, the interfacial polarization that results from the conductivity difference between the conductive GNS or GNS-PDA sheets and the insulating XNBR matrix is beneficial for increasing the dielectric constant. The GNS or GNS-PDA nano-sheets can be viewed as local micro-capacitors with GNS or GNS-PDA as electrodes and with a very thin host polymer layer in between as the dielectric [35]. In addition, after functionalization with PDA, the dielectric constant of the GNS-PDA/XNBR composite was higher than that of the GNS/XNBR composite. There are two reasons to explain the increased dielectric constant of the GNS-PDA/XNBR composite: (1) The GNS-PDA nano-sheets dispersion in the XNBR matrix is improved because of good interfacial interaction and this leads to a greater number of interfacial polarizations. (2) In the GNS/XNBR composite, GNS may connect with one another to form aggregations. This will minimize the number of micro-capacitors and cause leakage loss. However, the insulating PDA coated on the GNS makes the direct contact of GNS impossible and forms a segregated network. This structure increases the number of micro-capacitors in the GNS-PDA/XNBR composite and results in a larger dielectric constant [36,37]. The dielectric loss and AC conductivity at 100 Hz of pure XNBR and the XNBR composites are shown in Table 1. As seen in Table 1, the dielectric loss of the XNBR composites is a little larger than that of pure XNBR at 100 Hz. The increased dielectric loss of the XNBR composites is mainly attributed to the interface polarization (Maxwell-Wagner polarization) at the interface between the XNBR matrix and GNS nano-sheets. In addition, the AC conductivity of the XNBR composites is larger than that of pure XNBR at 100 Hz, which is ascribed to the conductive GNS nano-sheets.

**Figure 7:**
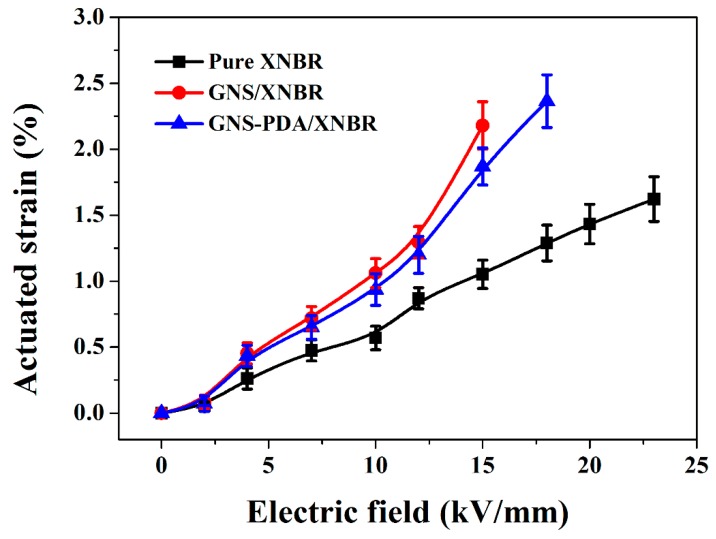
Dependence of actuated strain on electric field for pure XNBR, GNS/XNBR composite, and GNS-PDA/XNBR composite.

**Figure 8:**
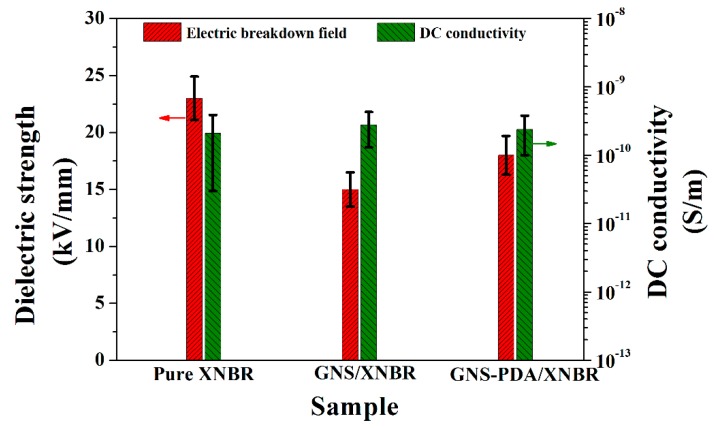
Dielectric strength and DC conductivity for pure XNBR, GNS/XNBR composite, and GNS-PDA/XNBR composite.

The effects of an electric field on the actuated strains of pure XNBR and the GNS/XNBR and GNS-PDA/XNBR composites are shown in Figure 7. It is clear that the actuated strains of the XNBR composites filled with GNS or GNS-PDA are much higher than that of pure XNBR at a certain electric field. The higher actuated strains are responsible for the small increase in the elastic modulus and large increase in dielectric constant of the XNBR composites with the addition of GNS or GNS-PDA nano-sheets, which leads to a larger value of β (Table 1). However, the actuated strain of the GNS-PDA/NBR composite is lower than that of the GNS/NBR composite at a certain electric filed. For example, the actuated strain of the GNS-PDA/NBR composite is 1.9% at 15 kV/mm, which is lower than that of the GNS/NBR composite (2.2%) at the same electric field. Although the dielectric constant of the GNS-PDA/NBR composite is higher than that of GNS/NBR composite, the elastic modulus of the GNS-PDA/NBR composite is also much larger than that of the GNS/NBR composite, and this leads to a lower value of β for the GNS-PDA/NBR composite (Table 1). According to the mechanism of DEAs, a high value of β leads to a high actuated stain at a certain electric filed. However, the GNS-PDA/XNBR composite has the largest dielectric strength of 18 kV/mm (Figure 8 and Table 1), which leads to it having the largest actuated strain of 2.4%, and this is much larger than that of pure XNBR of 1.3% at the same electric field (Table 1). 

The dielectric strength of pure XNBR and the XNBR composites are showed in Figure 8 and Table 1. It is clear that the dielectric strength of XNBR is decreased after introducing GNS or GNS-PDA nano-sheets, mainly because of the increase in DC conductance. Usually, a low DC conductance results in a high dielectric strength. This is often used as a tool to check the uniformity of an insulating material or to detect conductive impurities that affect the quality of such materials. This detection may not be possible using other methods [38]. The measured AC conductivity (σ_AC_) of any dielectric system is actually composed of two components according to the equation: σ_AC_ = σ_D_C__ + ωε”, where σ_D_C__ is the DC conductivity, ω is the angular frequency and ε” is the dielectric loss factor [34]. This equation suggests that with an increase in frequency, the contribution of the second part increases because of polarization toward an increase in total conductivity. In our work, dielectric strength was measured using an intelligent DC high voltage generator. In addition, the dielectric strength of the GNS-PDA/XNBR composite (18 kV/mm) is higher than that of the GNS/XNBR composite (15 kV/mm). There are four reasons that explain this phenomenon: First, the superior interface and better dispersion in the GNS-PDA/XNBR composite have fewer voids and defects than that of the GNS/XNBR composite: this leads to a higher dielectric strength. Second, the mobility of polymer chains is decreased because they are tightly bonded to the nano-filler via strong interfacial interactions. The transfer of charge carriers through loose polymer chains that are not bonded to the nano-filler is then blocked, and this results in a higher dielectric strength [39]. Third, the GNS are coated by an insulating PDA shell and show homogeneous dispersion in the XNBR matrix. The PDA shell prevents electron conduction and results in the lower leakage current and higher dielectric strength. Fourth, the EMI effect might be partially responsible for the decreased GNS/XNBR composite because the GNS/XNBR composite exhibits larger planar actuated strain than that of the GNX-PDA/XNBR composite at a given electric field [40,41]. When the voltage increases and exceeds a critical level, the membrane becomes thinner, and thus, the same voltage produces a higher electric field. The higher electric field further squeezes the membrane such that the elastic stress fails in equilibrium until there is an electrical breakdown. This failure mode is called electromechanical instability (EMI) or pull-in instability [40,41]. EMI is an important failure mode for the DEAs.

## 4. Conclusions

Improved mechanical and electromechanical properties of the XNBR based DEAs were achieved via the functionalization of GNS with PDA using a bio-inspired method and latex mixing. A segregated network structure of GNS-PDA filler was achieved in the GNS-PDA/XNBR composite via hydrogen bonds between –COOH groups of XNBR and phenolic hydroxyl groups of GNS-PDA that formed during latex mixing. Thus, the XNBR composite filled with GNS-PDA had improved filler dispersion and enhanced dielectric and electromechanical properties compared with the XNBR composite filled with pristine GNS. In addition, the insulating PDA coating on GNS leaded to the GNS-PDA/XNBR composite having a lower conductivity and a higher dielectric strength than those of the GNS/XNBR composite. Finally, the GNS-PDA/XNBR composite has an actuated strain of 2.4% with biaxial pre-strain of 10% at 18 kV/mm, and this value is much larger than the actuated strain of pure XNBR (1.3%) at the same electric field. This research provides a promising route for modifying high-dielectric-constant filler to improve mechanical and electrochemical properties of polymer dielectric composites.

## Figures and Tables

**Table 1:** Summary of electromechanical properties of pure XNBR and XNBR composites.

Sample	Dielectric Constant (100 Hz)	Dielectric Loss (100 Hz)	AC Conductivity (10^−11^ S/m 100 Hz)	Elastic Modulus (MPa)	β (MPa^−1^)	DC Conductivity (10^−10^ S/m)	Maximum Strain (%)	Breakdown Strength (kV/mm)
Pure XNBR	8.88 ± 0.14	0.606 ± 0.0051	3.06 ± 1.2	3.02 ± 0.19	2.94	2.1 ± 1.8	1.62 ± 0.17	23 ± 1.91
GNS/XNBR	10.15 ± 0.12	0.651 ± 0.0062	4.02 ± 1.6	3.34 ± 0.16	3.04	2.8 ± 1.5	2.18 ± 0.18	15 ± 1.53
GNS-PDA/XNBR	10.78 ± 0.11	0.647 ± 0.0056	3.17 ± 1.1	3.63 ± 0.14	2.97	2.4 ± 1.4	2.36 ± 0.20	18 ± 1.72

## Supplementary Materials

The following are available online at http://www.mdpi.com/2073-4360/11/2/218/s1, Figure S1: AFM height images of GNS.
