# Supplementary material for: Improved Mechanical and Electrochemical Properties of XNBR Dielectric Elastomer Actuator by Poly(dopamine) Functionalized Graphene Nano-Sheets
Source: Polymers (Basel). 2019 Jan 27;11(2):218. doi: 10.3390/polym11020218 (PMC6419049; doi:10.3390/polym11020218)
Supplement: Supplementary file 1 [file polymers-11-00218-s001.pdf]

Supplementary Information

Improved Mechanical and Electrochemical Properties of XNBR Dielectric Elastomer Actuator by Poly(dopamine) Functionalized Graphene Nano-Sheets

Dan Yang [1,2,*], Xinxin Kong [1,2], Yufeng Ni [1,2], Mengnan Ruan [2,3], Shuo Huang [1,2], Puzhen Shao [1], Wenli Guo [1,2], and Liqun Zhang [3,*]

[1] Department of Material Science and Engineering, Beijing Institute of Petrochemical Technology, Beijing 102617, China

[2] Beijing Key Lab of Special Elastomeric Composite Materials, Beijing 102617, China

[3] Department of Materials Science and Engineering, Beijing University of Chemical Technology, Beijing 100029, China

* Correspondence: yangdan@bipt.edu.cn (D.Y.); zhanglq@mail.buct.edu.cn (L.Z.)

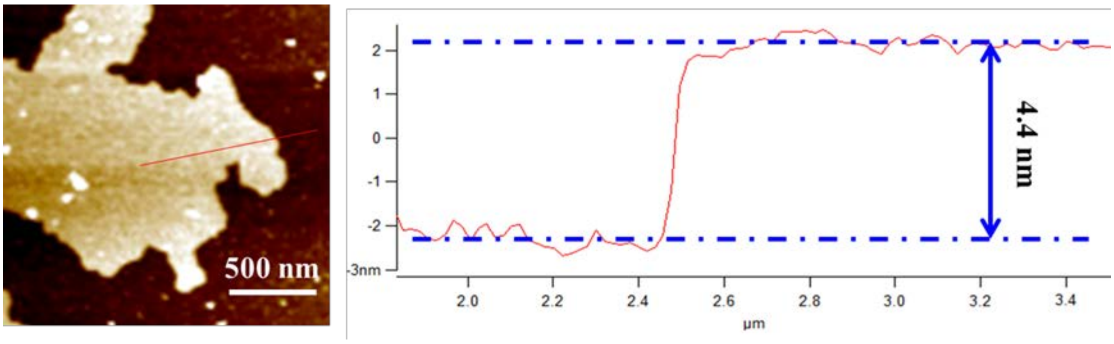

Figure S1. AFM height images of GNS.

The average thickness of GNS was determined by the MFP-3D Origin AFM (Oxford Instruments Co.; USA), which is shown in Figure S1. As shown in Figure S1, the average thickness of GNS is 4.4 nm.
